# Stricken Before Election: Presidential Health Crises From 1880 to 2020

**DOI:** 10.1097/AS9.0000000000000126

**Published:** 2022-01-11

**Authors:** Justin Barr, Theodore N. Pappas

**Affiliations:** From the Department of Surgery, Duke University Medical Center, Durham, NC.

**Keywords:** president, Chester Arthur, Teddy Roosevelt, Woodrow Wilson, Franklin Roosevelt, Dwight Eisenhower, Donald Trump, COVID-19, election

## Abstract

Between 1880 and today, 6 presidents have suffered major health crises just before their reelection campaigns. Ranging from Chester Arthur’s development of Bright’s Disease to Donald Trump contracting COVID-19, diseases and their treatments varied considerably. More interesting than the medical management, however, is the political maneuvering around each and the extraordinary lengths Presidents went to demonstrate their health to the American people. This article reviews these episodes, comparing and contrasting how each administration handled their crisis and what effect it had on the ensuing election—and thus the history of the United States.

## Abstract 2

Mini-Abstract: Between 1880 and 2020, 6 presidents have suffered major health crises just before their reelection campaigns, ranging from Chester Arthur’s development of Bright’s Disease to Donald Trump contracting COVID-19. This article reviews these episodes, comparing and contrasting how each administration handled their crisis and what effect it had on the ensuing election—and thus the history of the United States.

## INTRODUCTION

The announcement on October 2, 2020, that President Trump had contracted COVID-19, promptly followed by his hospitalization, shocked the world and upended an ongoing election campaign.^1^ It also raised the question, “has this ever happened before?” Indeed it has. History may not repeat itself, but it does rhyme: since the 1880s, 6 Presidents have experienced health crises before a national election. Chester Arthur developed Bright’s Disease; Theodore Roosevelt was shot in a failed assassination attempt; Woodrow Wilson suffered multiple strokes; Franklin D. Roosevelt’s heart disease significantly worsened; Dwight D. Eisenhower experienced a heart attack, followed by an operation for bowel obstruction; and, as noted, Trump was infected with COVID-19. Despite some gross similarities among these episodes, they are more interesting to examine for their differences—not only in the disease processes and management but also how the health crises were handled politically and their influence on the election that followed.

## CHESTER A. ARTHUR

Chester A. Arthur assumed office as the 21st president of the United States on September 20, 1881, following the assassination of President James Garfield.^2^ Early in his presidency, Arthur began experiencing fatigue. Dr. Brodie Herndon, Arthur’s brother-in-law, first recorded the President’s deteriorating health in February of 1882, noting that the President was “sick in body and soul,” possibly due to a complicated course of malaria.^3,4^ (Throughout the 19th century, malaria remained endemic to Washington, DC.)^5^ During the fall and winter of 1882, Arthur developed progressive fatigue, weight loss, anorexia, and peripheral edema. While the press continued to report that the President was ill from malaria, he was evaluated by several physicians for these additional symptoms and subsequently diagnosed with Bright’s disease. Roughly the modern equivalent of chronic kidney disease, Bright’s Disease (the first eponymously named condition) reflected contemporary understandings of progressive renal failure; despite efforts to elucidate the pathophysiology and natural history, there was no cure.^6,7^ Salem H. Wales, the former Surgeon General of the state of New York, diagnosed Arthur with Bright’s disease, later confirmed by a renowned contemporary physician in New York City, likely Alfred Loomis. Given popular knowledge of the poor prognosis associated with Bright’s Disease, the President and his physicians decided to keep the suspicion of this illness out of the public domain, going so far as to call it “pure fiction.”^8^

**FIGURE 1.**
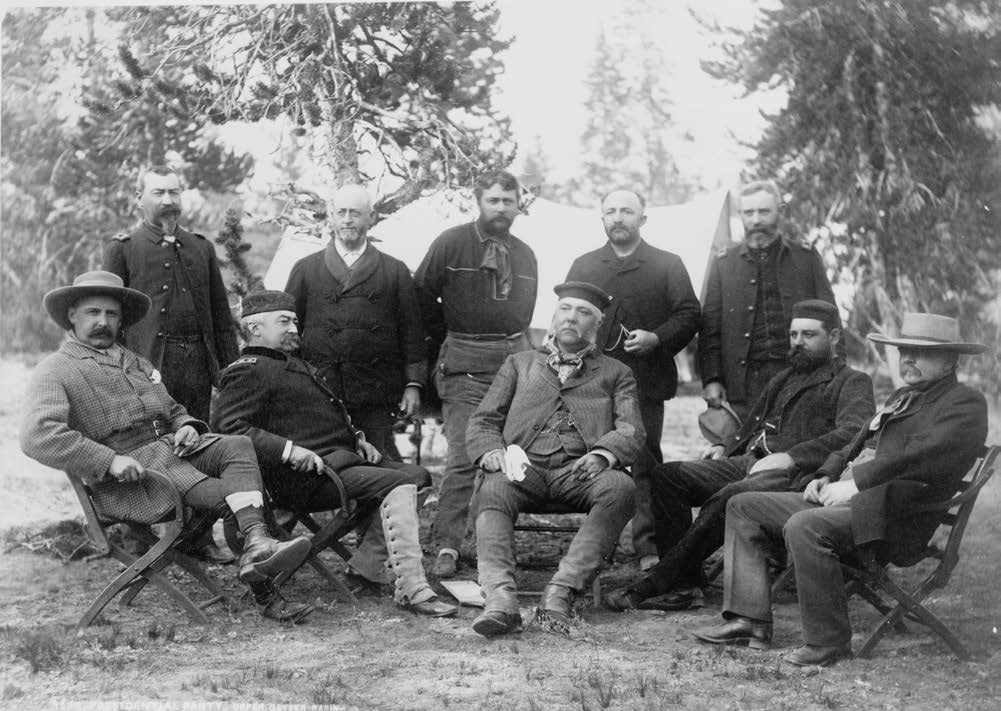
Chester Arthur visiting Yellowstone National Park (seated, center), Robert Todd Lincoln (seated to Arthur’s left), Lt. General Phillip Sheridan (seated, Arthur’s right). Photographer: F. Jay Haynes; image in the public domain through the Library of Congress (https://loc.gov/pictures/resource/cph.3c37259/, accessed October 17, 2021).

Throughout the final 2 years of his presidency, Arthur was noticeably ill, with episodes characterized by fluid retention, rigors, nausea, and colicky abdominal pain. He traveled to Florida and Yellowstone National Park, both to rejuvenate his health and simultaneously to prove his salubrity to a dubious press corps—an admittedly challenging combination (see Figure 1). As the 1884 presidential campaign approached, Arthur’s condition worsened. While not actively campaigning, which was common behavior for sitting presidents at the time, neither did he withdraw his name from consideration. After 4 rounds of voting during the national party convention, he lost the Republican nomination to the leading candidate, James G. Blaine of Maine, the sitting Secretary of State, a former US Senator, and also Speaker of the House. Blaine ultimately lost the presidential election to Grover Cleveland of the Democratic Party. It is unclear to what extent rumors of Arthur’s medical problems foiled his efforts at reelection, as hundreds of supporters voted for the sitting president, providing him the second highest delegate total in the first 3 presidential nomination ballots. Held in high regard by politicians, the media, and celebrities such as Mark Twain for his first term, an Arthur Republican ticket may well have defeated Cleveland in 1884.

After leaving office, Arthur worked in a New York law firm, but due to declining health he rarely conducted business, spending most of his time at home, often in bed. With a diminished appetite, he lost weight and experienced progressive weakness. The story of his Bright’s disease finally became public in June of 1885 with an article in the *New York Times*. In November, Arthur suffered a stroke and died a day later at the age of 57.

## THEODORE ROOSEVELT

Theodore Roosevelt, the 26th president of the United States, assumed office following the assassination of President William McKinley; Roosevelt was reelected in 1904. He declined to run in 1908, and William Howard Taft became president in 1909. When Taft ran for reelection in 1912, Roosevelt differed with him over a variety of policies including labor protections and tariffs. At the party convention in Chicago that summer, he challenged Taft for the Republican nomination. Taft ultimately was selected, and Roosevelt encouraged his delegates to walk out of the convention hall in protest.^9^

Having lost the Republican nomination, Roosevelt fulfilled his promise to campaign on a third-party platform. Now leading the Progressive “Bull Moose” Party, he ran against the Republican Taft and the Democratic candidate, Woodrow Wilson. On October 14, Roosevelt, while campaigning through the Midwest, was standing in his parked-car waving to well-wishers outside the Gilpatrick Hotel in Milwaukee, Wisconsin when John Schrank shot him at close range with a 0.38 caliber revolver.^10^ Schrank, a 36-year-old schizophrenic unemployed bartender, did not believe Roosevelt should run for a third term. George Washington had limited the number of presidential terms to 2 in his September 1796 farewell address, intending to prevent any American president from becoming a monarch like King George III.^11^ Roosevelt was the first president since Washington to challenge this unwritten term limit. Technically, Roosevelt had only been elected president once, his first term having started after the assassination of William McKinley. Still, a variety of presidential scholars and lay individuals (including Schrank) thought Roosevelt was defying a standard established by Washington and opposed his actions.

Fortunately, Schrank’s bullet passed through Roosevelt’s folded 50-page speech and careened off his metal glasses case before hitting his right chest. The bullet punctured the skin and subcutaneous tissue of the right chest but did not penetrate the pleural space.^12^ Interestingly, Roosevelt coughed into his hand after he was shot and noted there was no blood. He understood enough about chest injuries to know that hemoptysis following a chest wound suggested intrapleural penetration. His physician, Dr. Scurry L. Terrell, was traveling with the former President and recommended that he cancel the campaign event and proceed emergently to the local hospital. Roosevelt declined immediate medical care and asserted he was well enough to proceed to the Milwaukee Auditorium, where a large crowd awaited.^10^ He delivered his speech but after 84 minutes felt weak from blood loss and was driven to the Johnson Emergency Hospital in Milwaukee where he was met by local physicians. Colonel Roosevelt worried about the fragmented care that previous presidents (i.e., Garfield and McKinley) had received from teams of doctors.^2,13^ He wanted to transfer his care to Chicago where he thought the management might be more organized. Dr. Joseph Colt Bloodgood, visiting Milwaukee at the time, was also consulted about the ex-president’s injuries. Bloodgood had been William Halsted’s first chief resident and was exceptionally well-trained.^14^ He had strong family ties to the Milwaukee area where his brother, Wheeler P. Bloodgood, was a prominent lawyer and member of the Progressive Party who had made the local arrangements for Roosevelt’s trip. Dr. Bloodgood recommended that Roosevelt contact Dr. John B. Murphy of Chicago to assume his care.^15^ Murphy was a nationally prominent general surgeon noted for a variety of innovations including the right upper quadrant pain associated with cholecystitis, “Murphy’s sign.”^16^

**FIGURE 2.**
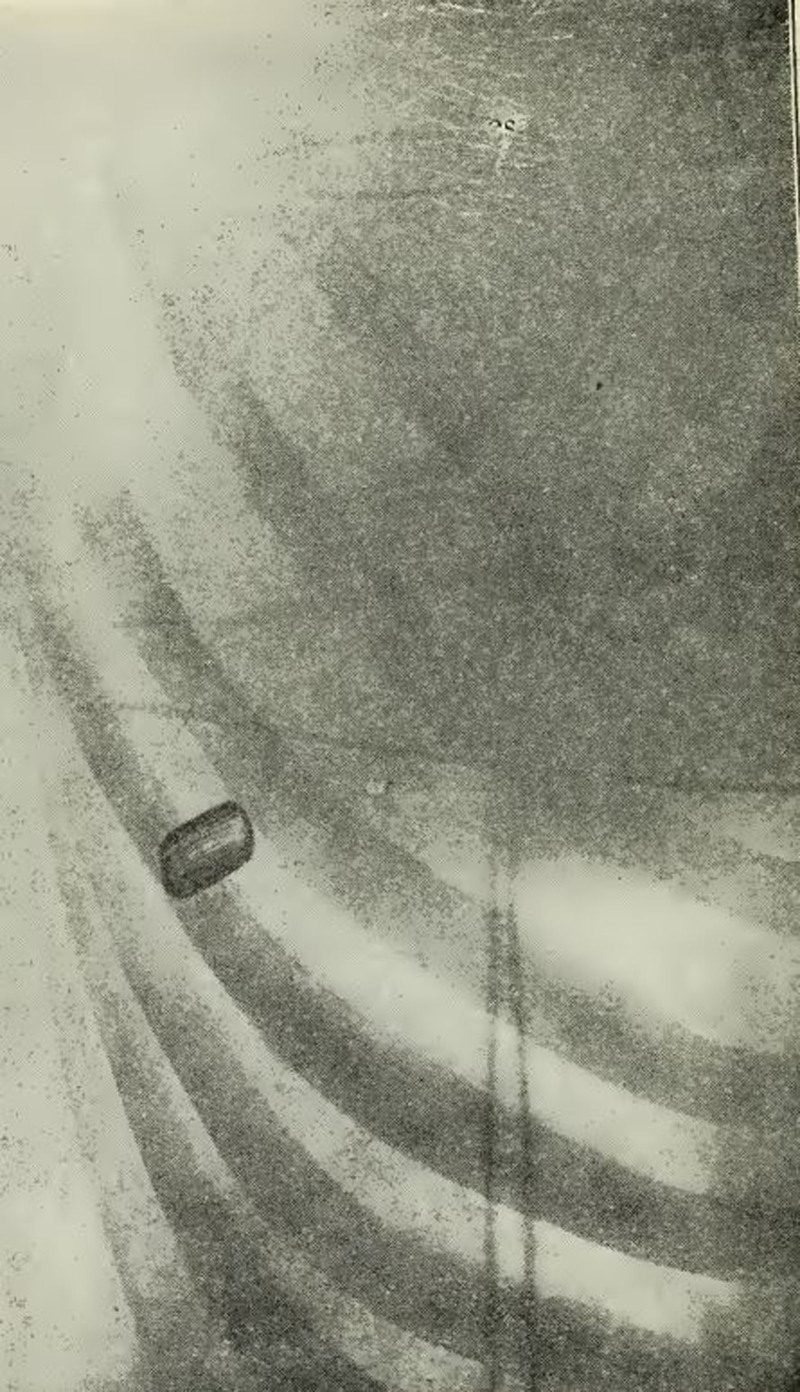
Chest roentgengram of the pistol bullet in Roosevelt’s left chest, taken by Dr. J. S. Jansseen at Johnston Emergency Hospital in Milwaukee.^15^

At 11:25 pm, Roosevelt was taken by car to the Chicago and Northwestern Depot in Milwaukee and boarded the train just after midnight on October 15. While Roosevelt sat in his private carriage, the “Mayflower,” the departure was paused as local surgeons reviewed his chest x-ray to determine if the former President was safe to travel. His train finally arrived in Chicago at 3:32 am where he was seen by Dr. Murphy at 5:12 am before disembarking. An ambulance pulled to the side of the Mayflower at 6:16 am, and Roosevelt was taken directly to Mercy hospital where he remained for 7 days.^10,15^ Murphy repeated a chest x-ray (Figure 2) that again demonstrated that the bullet injured the 4th rib and had lodged just outside the pleural cavity between the 4th and 5th ribs anteriorly.

During Roosevelt’s stay in the hospital, Woodrow Wilson and Howard Taft suspended their campaigns in deference to the former president’s injury.^17^ Roosevelt stated from his hospital bed on 17 October that he was ready to resume the race and encouraged Wilson and Taft to continue their efforts.^18^ Wilson officially restarted his campaign on October 27.^19^ Roosevelt did likewise later that month but never achieved his preinjury pace. He addressed large crowds in Madison Square Garden on October 30 and November 1, but polls continued to show Wilson leading both Roosevelt and Taft.^20^ Roosevelt eventually recovered his health but lost the 1912 election: he and Taft split the Republican vote allowing Wilson to win the election with 6,296,284 votes, which represented just over 41.8% of the voting electorate.^21^ Theodore Roosevelt lived the rest of his life in retirement without complication from the retained bullet in his chest wall.

## WOODROW WILSON

**FIGURE 3.**
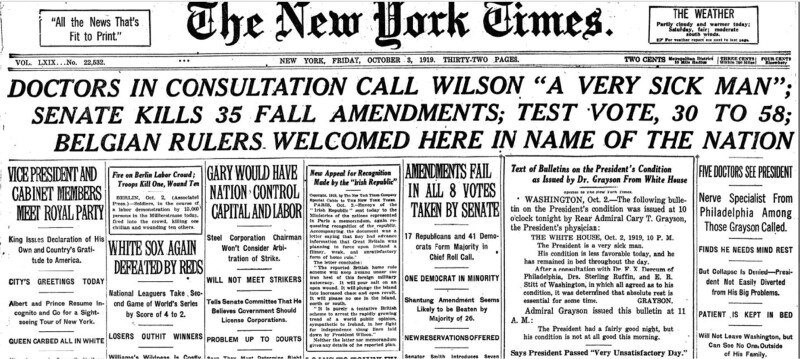
New York Times headlines announcing illness of Woodrow Wilson. Image in the public domain.

Having won the 1912 election, Thomas Woodrow Wilson served as the 28th President of the United States. When contemplating running for a third term in 1920, he was severely hindered by his neurologic condition. Wilson suffered a series of strokes before and during his presidency.^22^ The first stroke in 1896 presented as weakness in his right hand. In 1906, another left him blind in his left eye, and in 1913 a minor stroke affected his left arm. Most devastating was the stroke that occurred on October 2, 1919, leaving him paralyzed on the left side and with only partial vision in the right eye (Figure 3). Confined to his bed for several weeks, he never recovered sufficiently to resume his full duties as president. Wilson’s second wife, Edith, and his physician, Cary Grayson, continued to manage the presidency in his stead. The public’s awareness of Wilson’s disability in February 1920 prompted Republicans to call for someone in the administration to certify that the President was “unable to discharge the powers and duties” of the office, as required by the Constitution. However, at the behest of the First Lady, Wilson’s doctor would not publicly comment on the President’s health; this event preceded the passage of the 25th amendment that later allowed the vice-president and cabinet to assume presidential powers.

Despite his ongoing disability, Wilson not only served out his term but eagerly anticipated running for a third.^23^ Before the nominating convention, the President attempted to minimize his infirmity, so it would appear that his previous strokes would not prevent him from holding office again. In the spring of 1920, Wilson personally presided over cabinet meetings and took long rides in his car around Washington, DC, in an attempt to improve his public profile. Press releases and staged photographs touted his physical fitness. He entered the Democratic national convention in the summer of 1920 expecting party leaders to promote his candidacy. He planned to remain above the fray, then have party-elders nominate him when his competitors deadlocked, which was a reasonable strategy since no other leading candidate had emerged by July. Moreover, most Democrats supported Wilson’s policies, which aligned with the broader party platform. But Dr. Grayson rejected the idea of a third term for health reasons and communicated Wilson’s poor medical condition to party leaders before the convention.^23^ Ultimately, there were 22 ballots taken before the party nominated James M. Cox of Ohio as the candidate, paired with Franklin D. Roosevelt of New York as his running mate; Wilson’s name was never placed in nomination, largely due to his known condition. Cox was defeated by Warren G. Harding in the 1920 national election. Wilson opened a law practice in Washington, DC, in 1921, but he came to the office only once, and the practice subsequently closed a year later. He remained out of the public eye until his death on February 3, 1924, at the age of 67.

## FRANKLIN DELANO ROOSEVELT

Cousin of Teddy, Franklin Delano Roosevelt was elected in 1932 as the country’s 32nd president and remains the only person to have served more than 2 terms in office. Although paralyzed from polio throughout most of his political career, in his third term he also became profoundly ill from heart disease, a condition he, his physicians, and his advisers carefully guarded from the public to ensure victory in a fourth national election. W. Bruce Fye has recently published a compelling analysis of these events based on updated research in his book *Caring for the Heart.*^24^

On February 2, 1944, FDR was admitted to the National Naval Medical Center for the removal of a sebaceous cyst on the back of his head; the procedure was uncomplicated, but preoperative examination discovered severe hypertension. This finding prompted an evaluation by Dr. Howard Bruenn, a Johns Hopkins medical school graduate and noted heart-specialist in New York City who served in the Navy as Chief of Cardiology at Bethesda Naval Hospital during World War II.^25,26^ Bruenn noted a blood pressure of 186/108, arteriovenous nicking of the retinal arteries, cardiomegaly on chest x-ray, and cyanosis; an EKG demonstrated dramatic T-wave inversions, which by 1941 were well-known to signify severe heart disease with poor prognosis. Boston surgeon Frank Lahey and Atlanta internist James Paullin traveled to Washington, DC, to offer second opinions. Agreeing with Bruenn, together they recommended bed rest, a low salt diet, and digitalis, which Roosevelt accepted. Lahey, distressed about FDR’s health, wrote a secret memo documenting his concern that the president would not survive a fourth term.^27^

FDR’s personal physician, Dr. Ross McIntire, an ENT by training and Surgeon General of the Navy, deliberately mis-led reporters at the ensuing press conference on April 4, excluding any mention of hypertension or heart failure in what he called a “satisfactory” physical. Despite this report—and the interventions prescribed by Bruenn et al—FDR’s blood pressure remained high, *averaging* 209/100 in the 100+ measurements between the end of March and D-Day on June 6.^24^ Problematically in 1944, few effective treatments for hypertension existed.^28^ The President decamped to Bernard Baruch’s South Carolina estate in April and remained there for almost a month, returning to Washington refreshed but still ill. In July of 1944, he accepted the Democratic Party’s nomination for a 4th term of office, although he refused to campaign against his rival, Thomas Dewey, during the war.^27^ That summer and fall, rumors, leaks, and photographs showing weight loss led to national speculation over FDR’s health. Concerned that reports of sickness would doom Roosevelt’s reelection chances, his advisors scheduled a parade through New York City in October, a public event designed to showcase his salubrity—and hide his infirmity. Under the direction of J. Edgar Hoover, the Federal Bureau of Investigation interrogated—and intimidated—several Mayo Clinic physicians who had been discussing FDR’s cardiac problems. McIntire continued to lie blatantly at press conferences, asserting the President was healthy and able.^29^ Roosevelt ultimately won reelection with 53.4% of the national vote, his smallest margin of victory, and one that easily could have been swayed by credible reports of his ill health.

**FIGURE 4.**
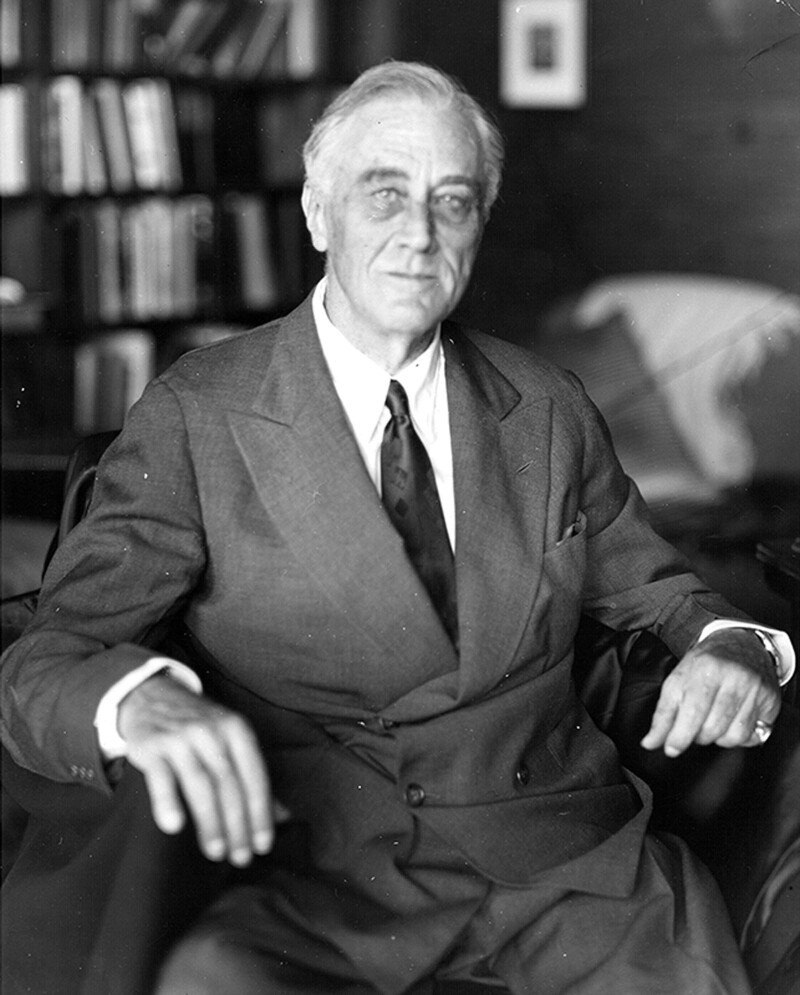
Last photograph taken of FDR, on April 11, 1945, by photographer Nicholas Robbins in Warm Springs, Georgia; FDR died the following day. Image from the Franklin D. Roosevelt Presidential Library & Museum and is in the public domain.

Throughout his fourth term, Roosevelt remained debilitated. His blood pressure on November 27, 1944 measured 260/150, prompting adjustments of his digitalis dose and more recommendations for rest. During his trip to the Crimean Conference in February, 1945 he developed pulsus alterans. By March, he had retreated to Warm Springs, Georgia for total rest. On April 12, Dr. Bruenn saw the President in the morning, who complained of a headache. In the early afternoon, his headache suddenly became severe (Figure 4). His blood pressure was 300/190, and he became unconscious, cold, and pale with wide dilation of his right pupil. Dr. Bruenn diagnosed a massive stroke and declared Franklin Delano Roosevelt dead at 3:35 pm on April 12, 1945.^24,25^

## DWIGHT D. EISENHOWER

**FIGURE 5.**
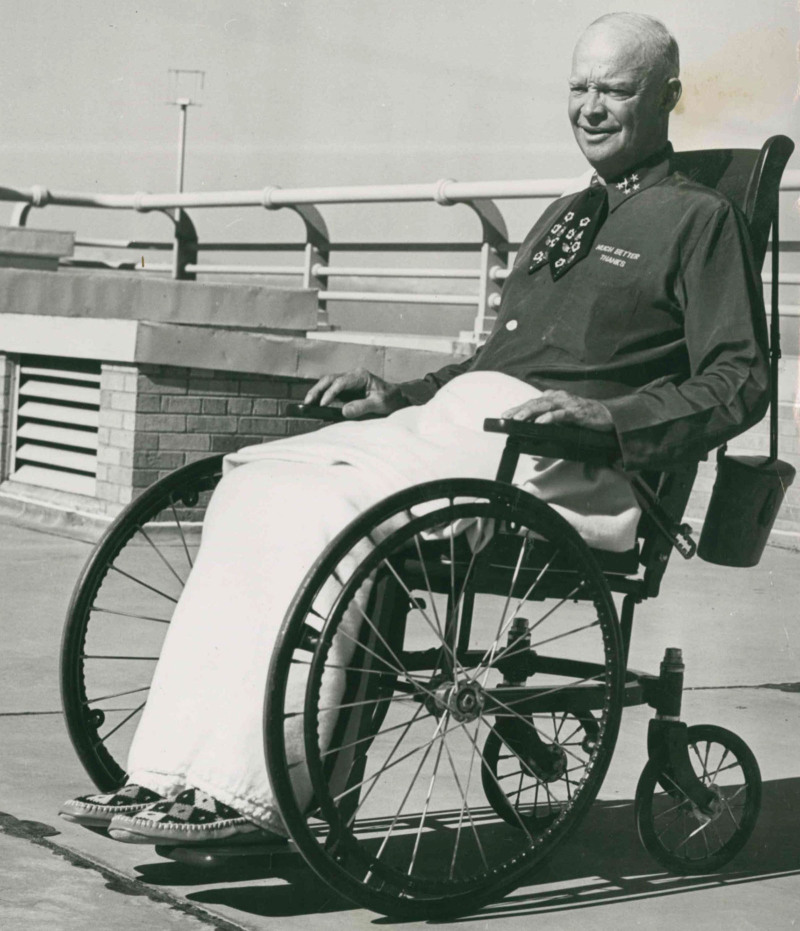
Ike on November 11, 1955, leaving Fitzsimmons Army Hospital. Photo from the National Archives, in the public domain.

Dwight D. Eisenhower was elected the 34th president of the United States in 1952. During his first term, he suffered 2 major illnesses that both affected his decision to run for a second term and, ultimately, his chances of winning: a heart attack in September 1955 and a laparotomy for bowel obstruction in June of 1956. The heart attack of 23 September 1955 started with “indigestion” on a Denver, Colorado golf course.^30,31^ The President’s physician Howard Snyder treated these symptoms conservatively until the following morning when it became obvious that the President was having a significant cardiac event. On Saturday, September 24, the President’s press secretary announced that Eisenhower had a “mild” coronary thrombosis and was being transported to Fitzsimmons Army Hospital. There he was treated by Colonel Thomas Mattingly MD, chief of cardiology at Walter Reed Army Hospital, and Paul Dudley White MD, a well-known Harvard cardiologist remembered today for elucidating Wolff-Parkinson-White syndrome. By Saturday afternoon, Eisenhower’s vital signs stabilized, and he was thought to be improving. Unfortunately, the President went on to extend his infarct and developed a ventricular aneurysm, although this setback was deliberately hidden from the public. The size of the initial infarction and the early extension of the ischemic area resulted in a very slow recovery (Figure 5). He remained in Denver at Fitzsimmons Army Hospital until November 11, insisting that he stay long enough to walk out under his own power rather than be ferried by wheelchair—having served under FDR, Ike was well attuned to the importance of appearing hale. After briefly staying in the White House, the President went to his Gettysburg, Pennsylvania home to convalesce further. It was the middle of December, 1955 before he again managed a full day of work in the White House. On February 12, 1956, after an extensive exam at Walter Reed, he was declared fit by his doctors, although both White and Mattingly recommended against him running for another term; White was slowly and gracefully dismissed from the medical team and Mattingly was kept under close observation at Walter Reed. Eisenhower decided to run for a second term while recuperating from his heart attack, although withheld a formal declaration of his intentions until a February 29, 1956, news conference to limit questions about reelection while in the throes of illness.^32^

This determination was premature, because in the spring of 1956 Eisenhower developed symptoms of abdominal pain and was diagnosed with regional enteritis.^33^ His pain worsened until 8 June 1956, when he was admitted to Walter Reed for bowel obstruction due to a Crohn’s disease stricture. He underwent exploratory laparotomy and ileal bypass surgery on June 9 by Leonard Heaton, the future Surgeon General of the Army, Isidor Ravdin, the chair of surgery at the University of Pennsylvania, and several others.^34^ Heaton was quick to assure the public that President did not have cancer. Postoperatively, Drs. White and Mattingly followed the President’s cardiac condition, which fortunately did not disrupt an uneventful recuperation. After discharge from the hospital on postoperative day 18, the President underwent further recovery at his Gettysburg home. Once again, he reaffirmed his fitness to run for a second term, a judgment reinforced by the conclusions of a panel of 9 physicians. Like many of his predecessors trying to prove their health, he traveled publicly to Panama (with his surgical drain in place) to demonstrate he had the stamina to fulfill another 4 years in office. Eisenhower recovered fully and ran for his second term, which he won in a landslide despite Adlai Stevenson’s attempts to question his fitness for office.

Eisenhower’s health problems did not end with reelection. He suffered a stroke of the middle cerebral artery on November 25, 1957, while still in office, from which he eventually made a full recovery. Repeated episodes of symptomatic cholelithiasis in 1966 led to an uneventful cholecystectomy in December of that year.^35^ His heart proved more problematic. Yet another myocardial infarction in 1965 curtailed his involvement in public affairs. He went on to have multiple heart attacks, ultimately leading to debilitating ischemic cardiomyopathy that required a lengthy admission to Walter Reed in 1968. His heart condition complicated a fourth and final laparotomy in February 1969 for bowel obstruction that also included a suprapubic catheter placement for prostatic hypertrophy. After spending almost a year in the since-named Eisenhower suite at Walter Reed Army Hospital, he died of congestive failure on March 28, 1969. Interestingly, an autopsy revealed a 1.5 cm adrenal pheochromocytoma that, in retrospect, may have partially explained his hypertension and heart disease.^36^

## DONALD TRUMP

In the fall of 2020, Donald J. Trump was running for president amid the international COVID-19 pandemic. On 1 October, a month before the general election, he developed respiratory symptoms and tested positive for COVID-19 on October 2. It is unclear where he acquired the disease, but Trump shunned wearing a facemask and had a variety of exposures to individuals with the disease in the preceding week.^37,38^ Just a few days prior, 150 mostly mask-less individuals gathered at the White House for the announcement of the President’s Supreme Court selection, several of whom later tested positive, including Hope Hicks, one of the president’s closest advisors.^39–41^ Recent revelations by Mark Meadows, his chief of staff, disclosed a positive test on September 26, 2021, before multiple public events hosted by the president, who reportedly tested negative thereafter.^42^ The President’s first known symptoms appeared during a fundraising trip to the Trump National Golf Club at Bedminister, New Jersey on October 1. Fatigued with a cough and nasal congestion, Trump tested positive upon returning to the White House that evening via a rapid test, later confirmed by PCR that same night. The First Lady, Melania Trump, simultaneously became symptomatic, with subsequent confirmation that she too had contracted COVID-19.

News of the President’s test results came at 12:54 AM, October 2 via the President’s personal Twitter account—interestingly, his most “liked” Tweet to date.^43^ On Friday, October 2, the Marine One helicopter transported him to Walter Reed National Military Medical Center upon the recommendation of his physician, Dr. Sean Conley. Conley had obtained his doctorate from the Philadelphia College of Osteopathic Medicine in 2008 and served as a trauma surgeon in Afghanistan; he functioned as the spokesperson for Trump’s medical team that included a number of intensive care and infectious disease physicians. At the time, Trump was reportedly suffering from increasing upper respiratory symptoms, associated with a rising fever and worsening hypoxia, a condition that demanded supplemental oxygen.^44^ After admission, Trump was given Regn-cov2(8-gram dose), a monoclonal antibody preparation from Regeneron Pharmaceuticals that had yet to be approved by the FDA but was administered after a “compassionate use” request by the Walter Reed physicians. That same night, Trump was given the antiviral drug Remdesivir.^45,46^ While in Walter Reed, the President’s hypoxia worsened, requiring supplemental oxygen. He was treated with steroids (dexamethasone) on October 4 after another episode of hypoxia.^47,48^ Anxious to demonstrate his health to a worried American public (and electorate), Trump defied the recommendations of public health experts to take a motor-tour around Walter Reed National Medical Center. Widely criticized at the time, this publicity stunt exposed his Secret Service detail to the virus while sitting with Trump in his hermetically sealed presidential limousine.^49^ On regular doses of dexamethasone and Remdesivir, the President’s condition improved, and he was discharged at 7:00 pm on October 5.^50^ He continued to receive doses of Remdesivir and Regeneron after returning to the White House.^46,51,52^ On October 10, Dr. Conley stated that the President was no longer a transmission risk, as defined by undetectable subgenomic mRNA and a resolution of symptoms.^53^

After being discharged from the hospital, Trump restarted campaigning. He made his first public comments on October 10 and spoke to large crowds through the latter half of October.^54,55^ Polls, however, continued to forecast Trump trailing the Democratic nominee Joe Biden by at least 7 percentage points through the beginning of November.^56^ Biden had considerately slowed his campaigning during Trumps hospitalization in deference to the illness, in particular suspending ads that were viewed as “negative” on October 2.^57^ He also decreased his live events, but this was likely due to safety issues concerning the spread of COVID 19.^58^ The national election took place on November 3, and Biden received 51.3% of the popular vote and totaled 306 electoral votes to Trump’s 232 electoral votes.

## CONCLUSIONS

Between 1880 and 2020, at least 6 presidents suffered severe health crises before a national election. Comparing and contrasting these incidents reveals interesting themes worthy of exploration, most centered around the question: how, if at all, did the various pathologies affect the subsequent voting (and in so doing, the history of this nation)? The 140-year gap included substantial medical advances: the Bright’s Disease that plagued Arthur and the hypertension that contributed to FDR’s death are both treatable conditions in 2021 that would be unlikely to influence chances of political victory today. Conversely, Wilson’s severe poststroke disabilities remain challenging to manage despite the technological and scientific improvements of the last century. The nature of the malady and the degree to which the patient was perceived to be at fault also factored heavily. At one end of the spectrum, Teddy Roosevelt, already a war hero, was shot by an assassin, a condition not just blameless but one that likely boosted his public image. On the other hand, many Americans felt Trump, with his willful disregard of public health measures such as facemasks and his insouciant approach to the pandemic generally, bore some responsibility for his condition, and that the illness epitomized a failure of leadership in controlling the epidemic. FDR, Wilson, and Arthur fall somewhere in the middle, suffering conditions relatively blameless but also recognized by contemporaries as untreatable and eventually fatal.

While therapeutic strategies differed substantially for the various conditions over the years, it is interesting to note that most presidents sought out nationally reputed experts to treat their ailments. Arthur turned to a trusted family member as well as famous doctors in New York City. Teddy Roosevelt relied on Bloodgood and Murphy, 2 of the most accomplished and highly regarded surgeons of the day. His cousin FDR called upon an authority in heart disease to manage his hypertension on a daily basis while also consulting experts such as Frank Lahey for second opinions. Ike had both world-renowned cardiologists and surgeons guiding his care. All these presidents looked beyond both government/military circles and their existing general practitioner to seek outside, authoritative advice. Exceptions included Wilson, who relied on his established physician for a condition well-known to be untreatable, and Trump, whose staff physician led a team of on-call doctors at the military hospital, perhaps reflecting his distrust of the elite medical establishment.

Tellingly, every president sought to hide his infirmity outright or otherwise minimize its severity. Arthur and Wilson both went to great lengths to conceal their diagnoses while embarking on public tours to demonstrate their fitness. Teddy Roosevelt, publicly shot, nonetheless delivered a speech that evening to prove his robust health was unaffected by a mere flesh wound; he repeatedly reassured the American people that he was fine and fit to serve. His cousin FDR, already debilitated by polio, was sensitive as to how the press wrote about—and how Americans thought about—his health. He took extraordinary measures to veil his heart disease from the public, with his personal physicians blatantly lying to the press and the FBI intimidating physicians into silence. Secrecy around World War II in general abetted FDR’s suppression. By the latter half of the 20^th^ century, changes in media, technology, and general invasiveness into the private lives of public figures has made such masking increasingly difficult. Whereas presidents before 1950 could effectively hide their condition, those after 1950 instead focused on managing its presentation to the public. Both Eisenhower’s heart attack and bowel surgery were common knowledge with extensive press coverage. But Ike made sure to grant personal interviews to reporters to demonstrate his recovery, seeking to dispel any thoughts of being unable to serve a second term. He made repeated public appearances and even delayed his hospital discharge until he could walk in order to demonstrate his full recovery. Political Scientist Robert Gilbert has documented how Eisenhower carefully scripted accounts of his illnesses and manipulated events to improve his chances at reelection.^31^ Trump allegedly hid his first positive COVID test results for days before announcing his infection on Twitter after his condition deteriorated to where he assumed the news would soon leak online. Like his predecessors dating to Arthur, he took a public-relations tour around Washington DC to showcase his health; unlike his forebearers, Trump was still infected with a contagious disease. Subsequent reports indicated that his team minimized the severity of his condition to reassure Americans—and prospective voters.^59,60^

These efforts raise questions about the extent of medical privacy that is appropriate for public figures, a topic Baron Lerner has addressed in some depth.^61^ In America, citizens expect to know about conditions that might affect the ability of a candidate to fulfill his or her duties, particularly in such a critical role as President of the United States. Presidents, however, repeatedly demonstrated that they did not believe the public necessarily had a right to the truth about their health, evidenced by their robust efforts to hide information and/or downplay their illness. This deception appears to rise more from political calculations and concerns regarding electability rather than any abstract or personal notion of privacy, which complicates traditional bioethical arguments. Despite attempts to conceal information, there is no evidence to suggest these presidents refused treatment or received suboptimal care in the name of secrecy.

Ultimately, it is impossible to determine to what extent these health crises affected any Presidential election. Every national contest depends on variables ranging from party appeal to contemporary political issues to reputation of the candidates. Still, it seems highly likely that Arthur did not receive his party’s nomination due to his suspected/known diagnosis of Bright’s Disease, with its terminal prognosis, thereby diminishing his party’s chances to retain the office. Teddy Roosevelt faced a nigh-impossible hurdle as a third-party candidate; his attempted assassination and campaign pause probably did not affect the end-results. Wilson also was a long-shot to break the traditional 2-term limit in 1920; his infirmity scuttled any chance he had. FDR clearly won despite suffering from severe heart disease, although his advisors were absolutely convinced that public knowledge of his poor health would doom the campaign. This fear led to extraordinary efforts at subterfuge. Similarly, Ike won reelection despite the heart attack and bowel operation but relied on physicians reassuring the American people of his good health. Broad popularity among Americans in 1952 also facilitated Ike’s victory. Trump’s loss in the 2020 election is one historians will analyze for years to come. Although he trailed in the polls before his COVID-19 infection, later results proved these tallies largely inaccurate. Trump connected best with voters in large rallies; his inability to do so in the waning months of campaign season (due to his own illness and the national pandemic) may well have contributed to defeat.

As politicians enter office ever-older, future presidents will likely continue to suffer illnesses, some inconveniently timed around their reelection. History suggests they will seek to hide, minimize, and trivialize these conditions in an effort to convince the American people of their fitness for office. This desire stems from Americans insisting on super-human status from their politicians, perhaps based on the ill-founded belief that presidents are supposed to be (or appear to be) indestructible representatives of the country. Elucidating a history acknowledging otherwise may relieve some of this pressure and lead to more honest conversations concerning presidential health and electability.
